# Identification of stress-alleviating strains from the core drought-responsive microbiome of *Arabidopsis* ecotypes

**DOI:** 10.1093/ismejo/wraf067

**Published:** 2025-04-09

**Authors:** Zewen Li, Zhenghong Wang, Yujie Zhang, Jianbo Yang, Kaixiang Guan, Yi Song

**Affiliations:** Shenzhen Key Laboratory of Plant Genetic Engineering and Molecular Design, Institute of Plant and Food Science, Department of Biology, School of Life Sciences, Southern University of Science and Technology, Shenzhen 518055, China; Shenzhen Key Laboratory of Plant Genetic Engineering and Molecular Design, Institute of Plant and Food Science, Department of Biology, School of Life Sciences, Southern University of Science and Technology, Shenzhen 518055, China; Shenzhen Key Laboratory of Plant Genetic Engineering and Molecular Design, Institute of Plant and Food Science, Department of Biology, School of Life Sciences, Southern University of Science and Technology, Shenzhen 518055, China; Yunnan Key Laboratory for Wild Plant Resources, Department of Economic Plants and Biotechnology, Kunming Institute of Botany, Chinese Academy of Sciences, Kunming, Yunnan 650201, China; Shenzhen Key Laboratory of Plant Genetic Engineering and Molecular Design, Institute of Plant and Food Science, Department of Biology, School of Life Sciences, Southern University of Science and Technology, Shenzhen 518055, China; Shenzhen Key Laboratory of Plant Genetic Engineering and Molecular Design, Institute of Plant and Food Science, Department of Biology, School of Life Sciences, Southern University of Science and Technology, Shenzhen 518055, China

**Keywords:** drought, core microbiome, natural ecotypes, transcriptome, stress-alleviating microorganisms

## Abstract

Plant genetic and metabolic cues are involved in assembling their “core microbiome” under normal growth conditions. However, whether there is a core “stress responsive microbiome” among natural plant ecotypes remains elusive. Drought is the most significant abiotic stress worldwide. Characterizing conserved core root microbiome changes upon drought stress has the potential to increase plant resistance and resilience in agriculture. We screened the drought tolerance of 130 worldwide *Arabidopsis* ecotypes and chose the extremely drought tolerant and sensitive ecotypes for comparative microbiome studies. We detected diverse shared differentially abundant ASVs, network driver taxa among ecotypes, suggesting the existence of core drought-responsive microbiome changes. We previously identified 1479 microorganisms through high-throughput culturing, and successfully matched diverse core drought responsive ASVs. Our phenotypic assays validated that only those core drought responsive ASVs with higher fold changes in drought tolerant ecotypes were more likely to protect plants from stress. Transcriptome analysis confirmed that a keystone strain, *Massilia* sp. 22G3, can broadly reshape osmotic stress responses in roots, such as enhancing the expression of water up-taking, ROS scavenging, and immune genes. Our work reveals the existence of a core drought-responsive microbiome and demonstrates its potential role in enhancing plant stress tolerance. This approach helps characterize keystone “core drought responsive” microbes, and we further provided potential mechanisms underlying *Massilia* sp. 22G3 mediated stress protection. This work also provided a research paradigm for guiding the discovery of core stress-alleviating microbiomes in crops using natural ecotypes (cultivars).

## Introduction

Higher plants are sessile organisms and thus frequently affected by numerous biotic and abiotic stresses. Meanwhile, higher plants are also surrounded by an enormous amount of commensal microbes, which serve as the second genome of the host [1, 2]. Among those microbiome members, core microbiomes usually refer to the microbial taxa shared among two or more genotypes [3, 4], or microbes that are central to community network structure or interactions [5]. It has also been reported that core microbiome can be effectively used in agroecosystem applications [6, 7]. Utilizing natural variations of plant ecotypes or human cohorts [4, 8, 9], comparative microbiome studies have confirmed that higher eukaryotes have conserved core microbiome composition under normal conditions. However, it remains unknown whether hosts have evolutionarily conserved core stress-responsive microbiome changes. Considering that higher plants shared substantial similarities in the core biotic (e.g. immune systems) and abiotic (e.g. hormone pathways) stress responsive genetic framework [10, 11], it is plausible that different plant ecotypes might also share some conserved “core stress responsive microbiomes”. Understanding the “core stress responsive microbiome” can help identify major keystone stress-alleviating microbes, which would also lay the foundation for in-depth studying of root-microbe interaction mechanisms under stresses.

During long-term plant-microbiome interactions, certain plant associated microbiome can be shifted to a stress-alleviating microbiome upon specific stresses [12, 13]. This phenomenon is called plants mediated “cry for help” to the microbiome [14], and was initially observed in disease suppressive soils [15]. At least two essential criteria are needed to confirm the existence of “cry for help” process: (i) there are genetic or metabolic evidence supporting host effects are involved in affecting microbiome composition upon stresses [16, 17]; (ii) the shifted microbiome confers causal growth promotion or enhanced stress tolerance for the host [15, 18]. We previously identified a plant receptor-like kinase regulator that selectively regulates beneficial *Pseudomonas* colonization in roots, providing a genetic evidence that host can potentially “cry for help” to beneficial microbes [16]. However, the role of plant genes in modulating drought-induced microbiome changes and the potential consequences of these changes are less studied.

Drought is a major abiotic stress worldwide exacerbated by global climate change [19, 20]. Drought drastically affects both plant and microbe growth [19, 21, 22], and further systematically disturbs the composition [23], maturity and development [24, 25], network interactions and stability [26, 27] in root associated microbiomes. Numerous studies suggest that drought can trigger the conserved enrichment of drought-alleviating Actinobacteria (especially the genus *Streptomyces*) in multiple plant species [21, 24, 28]. Moreover, emerging evidence supports that other drought-alleviating microbes could also be enriched upon drought stress, including *Rhizobiaceae* [29], *Acinetobacter*, *Pseudomonas* [30], and *Curtobacterium* [31]. Those studies support the existence of broad “cry for help” phenomenon to diverse beneficial microbial taxa during drought stress.

To date, we still know little about whether plant genes are involved in “cry for help” to regulate beneficial microbiome changes upon drought. Drought broadly affects photosynthesis [32], hormone signaling, root architecture [33], and root exudate compositions [25, 34, 35] in plants, which indicates that plant might be involved in orchestrating microbiome changes upon drought. Our recent work provided genetic evidence that mutants of root hair developmental regulators affecting the abundance and network interactions of stress-alleviating microbes [29]. However, whether different plant ecotypes or cultivars show conserved “core drought responsive microbiome” changes remain unknown.

Natural plant ecotypes (or cultivars) provide ideal genetic variation populations to address the fundamental questions related to “core microbiome” compositions. Pioneering studies using different natural *Arabidopsis* ecotypes have characterized the conserved microbiome compositions in both root and rhizosphere microbiome [4, 8]. These studies strongly suggested that plant roots can assemble a core microbiome distinct from the surrounding bulk soil. A screening of 196 geographically representative natural *Arabidopsis* ecotypes suggests that different ecotypes harbor different levels of beneficial fluorescent *Pseudomonads* [36], and beneficial *Pseudomonas* strains only exhibit compatible growth-promoting effects in ecotypes that can support high levels of *Pseudomonas* colonization. This indicates that different natural *Arabidopsis* ecotypes have specific strategies for interacting with beneficial microbes. Furthermore, natural *Arabidopsis* ecotypes have not been genetically selected during the breeding process, and thus, they are expected to have more naturally occurring genetic variations without artificial selection. These elegantly selected representative *Arabidopsis* ecotypes provide valuable materials to investigate whether natural host genetic variations assemble convergent or divergent microbiome structures under stresses.

In this study, we screened a total of 130 natural *Arabidopsis* ecotypes (from 196 geographically representative natural *Arabidopsis* ecotypes collections [36]) for their drought tolerance in natural soil harvested from tropical rainforest and dry-hot valley ecosystems. We hypothesized that: (i) There might be conserved “core drought responsive” microbiome members among diverse ecotypes; (ii) Extremely drought tolerant and sensitive ecotypes might show differences in their interactions with root microbiomes upon drought stress; (iii) Characterizing “core drought responsive microbiome” among different ecotypes might help us predict the keystone drought-alleviating microbes. Thus our objectives were: (a) to investigate whether natural ecotype variations share a similar or core stress-responsive microbiome under drought; (b) to predict and identify keystone microbes that enhance plant stress tolerance; (c) to dissect the potential molecular pathways underlying microbes mediate stress alleviation in plants.

## Materials and methods

### Natural soil collection

The natural soil used in the present study was collected from the Xishuangbanna Tropical Botanic Garden (E101°27′, N21°92′), and the Yuanjiang Savanna Ecosystem Research Station (E102°10′, N23°28′) of the Chinese Academy of Sciences, separately. The top 3 cm, including visible stones and leaf litter, was cleared before harvesting. After that the next 10 cm of soil were collected. The collected soil was then transferred to the lab and sieved through a 2 mm sieve to further remove the large particles such as plant debris and soil protozoa.

### Plant materials collection and growth conditions

All the *Arabidopsis* seeds were sterilized with chlorine gas (exposure to 100 ml bleach plus 5 ml concentrated hydrochloric acid) for 20 min. Seeds were then soaked in a sterilized 0.1% agar solution and stored at 4°C in the dark for 48 h. Seeds were germinated on the 1/2 Morishige and Skoog (MS) agar plates containing 1% sucrose. Seedlings on the plates were grown in a growth chamber (Fujian JIUPO, BPC500 H), at 22°C under a 16 h light/ 8 h dark cycle for 7 days before transplanting.

The natural soil from the two locations was thoroughly homogenized before use. To improve plant health, the natural soil was mixed with other soil substrates (2:2:1:1 ratio of commercial growth soil, vermiculite, perlite, and natural soil). The soil was scooped into 6 × 6 cm pots (four plants per pot) for *Arabidopsis* growth. Plants were grown under a cycle of 10 h days and 14 h nights with a light intensity of 100 μmol m^−2^ s^−1^.

### Drought treatment

Seven-day-old seedlings were transplanted from plates into the pot (6 × 6 cm) that was filled with experimental soil (4 plants/pot). After a well-watering management period of 14 days, plants of each ecotype were randomly assigned to either the control or drought treatment. The plants in the control group were managed with a normal watering regime, which was watered every 4 days (700 ml/per tray). In contrast, the plants in the drought treatment group were completely stopped watering at the 14^th^ day after transplantation into soil. All the pots were randomly rotated every day to avoid uneven drought treatment.

### Harvesting root microbiome samples

The plants of each ecotype per treatment were individually scooped out of the pot, and the soil loosely adhering to the roots were removed by gently shaking. Roots were immediately cut and harvested. After being cleaned in the Falcon tube filled with 25 ml of sterilized PBS (10 mmol/L) three times, the roots were transferred into a new Falcon tube containing fresh PBS. They were then washed and sonicated (at 40 Hz for 10 s) to further remove loosely attached root surface soil. Seven pots (four plants in each pot) were grown for each ecotype within each treatment, four plants from different trays were mixed as a replicate. We also prepared pots without plants but under the same environmental conditions for sampling bulk soil samples. The top 2 cm of soil had been removed, and then four sites were harvested and mixed from each pot as a bulk soil sample. Finally, four biological replication samples were collected for each ecotype within each treatment for microbiome sequencing. All the microbiome samples were stored at −80°C prior to use.

### Microbiome sample preparation, sequencing, and data processing

DNA for microbiome sequencing was extracted using the Power Soil DNA Isolation Kit (Qiagen, Germany). The V3-V4 regions of the bacteria 16S rRNA genes were then amplified using the primers 349F (5’-ACTCCTACGGGAGGCAGCA-3′) and 806R (5’-GGACTACHVGGGTWTCTAAT-3′). Finally, paired-end 250 bp sequencing was performed on a NovaSeq 6000 System (Illumina). The 16S rRNA gene amplicon sequencing data were processed using QIIME2 v.2022.2 [37]. Briefly, the DADA2 pipeline was used to generate the amplicon sequence variants (ASV) [38]. The taxonomic annotation of each ASV was classified using SILVA database (release 138) with a pre-trained out Bayes classifier [39, 40]. ASVs assigned to chloroplast and mitochondria were considered as contaminations and were removed. Furthermore, ASVs that occurred only in one sample were also removed. The retained ASVs were then used for downstream analysis.

### Network analysis

ASVs with relative abundance greater than 0.2% within the root samples of each ecotype and occurring in at least two samples were used for the construction of the microbial community network. Briefly, spearman correlation analysis was performed using *ggClusterNet* [41]. Correlations between paired ASVs with a coefficient greater than 0.9 and an FDR-adjusted *P* value below 0.05 were retained. Topological parameters of each network, such as counts of nodes and edges, as well as the node properties like degree and betweenness, were computed using the *igraph* package in R [42]. The NetShift analysis was performed to identify potential keystone driver genera based on the changes in network interactions of the microbial community network from the control and case (drought) treatment [43]. This tool allows quantification of the interaction changes of individual nodes (taxa) between the drought and control group, and it determines whether their importance, based on neighbor shift score, increases in the drought samples.

### Isolation and identification of root-associated microbes

Root-associated microbes were isolated according to previously published methods [44]. Briefly, the roots of *Arabidopsis* under both drought and well-watered conditions were selected. The roots were harvested from the soil and washed three times with sterilized PBS solution (0.1 mol/L). Cleaned roots were then cut into 2 mm pieces, and 0.2 g of root tissue were weighed and placed into a sterilized tube filled with 200 μl of sterilized MgCl_2_ (10 mmol/L). The mixture of tissue and solution was ground into a homogeneous slurry. The slurry was then transferred into a new sterilized tube filled with 25 ml sterilized MgCl_2_ (10 mmol/L) and thoroughly mixed. Different volumes of mixture were separately transferred into different bottles, each containing 1 L of 10% TSB (tryptic soy broth), R2A, and ISP (international Streptomyces liquid) medium. These mixtures were then further diluted using gradients of 222×, 666×, 2000×, 6000×, 18 000×, and 54 000×. The three appropriate volumes, representing specific dilution folds of the mixture, were then confirmed using the methods described in ref [44]. 160 μl of diluted media was distributed into a 96-well plate and incubated for 14 days at 28°C. The plates with ~30% of wells showing bacteria growth were selected. The bacteria culture solution in each well from these plates were carefully transferred into new 96-well plates and uniquely numbered. We then added 120 μl of glycerol to each well. All the plates were then stored at −80°C prior to use.

For the identification of the isolates, we first extracted the DNA of the isolates using the MagaBio plus bacteria Genomic DNA Purification Kit (Bioer, Hangzhou, China). To better align with the differential ASVs during the analysis of 16S rRNA gene amplicon sequencing data from root-associated samples, the V3-V4 region of the 16S rRNA gene of these isolates were amplified using the same primers, 349F (5’-ACTCCTACGGGAGGCAGCA-3′), and 806R (5’-GGACTACHVGGGTWTCTAAT-3′). Library were then constructed using NEBNext Ultra II DNA Library Prep Kit (New England Biolabs, USA). The Qubit 4.0 Fluorometer (Thermo Scientific) was then used to determine the quality of the library. Paired-end 250 bp sequencing was performed on a NovaSeq 6000 System (Illumina). The sequences of all isolates were deduplicated using VSEARCH v2.15.2 [45] initially. The unique sequences were then clustered into operational taxonomic units (OTUs) at 97% similarity using UPARSE [46]. The representative sequence of each OTU (unique isolate) was taxonomically annotated using USEARCH11 based on 16S SILVA v138 database. The unannotated sequences in the SILVA database were further aligned to the NCBI database with a similarity greater than 97%. The unique bacteria strains were then stored in sterile 20% glycerol and kept at −80°C.

### Plant growth promotion assay of isolates on polyethylene glycol plates

To match differential abundant ASVs and our isolates, we aligned the marker sequence derived from our isolated ASVs to the 16S rRNA gene sequence database of all isolates. The reference database, consisting of 16S rRNA gene sequences of all isolates, was created using BLAST v.2.12.0 with the *makeblastdb* function [47]. The polyethylene glycol (PEG) plates-based osmotic stress treatment system was used to assess the effects of each selected strain on plant performance under drought conditions [48, 49]. For PEG treatments, 5-day-old seedlings of Col-0 from 1/2 MS plate (containing 1% sucrose) were transferred to either control plates (1/2 MS containing 0% sucrose) or 25% PEG plates. To make PEG plates, the filtered solution of PEG overlay (no agar) containing 1/2 MS, 6 mmol/L MES, and 25% PEG8000 (250 g per litter) were added on top of normal 1/2 MS plates. The plates with overlay were allowed to equilibrate for 24 h, after which the overlay solution was removed. For each type of plate (control or PEG), a total of 2 μl strain culture (OD_600_ = 0.005) and the same volume of 10 mmol/L MgSO_4_ (mock) was separately inoculated onto the root surface of the plants. We recorded the primary root length and shoot fresh weight at 14 and 21 days after germination, respectively.

### RNA sequencing and data analysis

For RNA-seq, plants grown on 1/2MS plates and PEG plates were harvested 9 days after inoculation with or without *Massilia* sp. 22G3. Total RNA from roots samples was extracted using TRIzol (Bio-Rad, USA) reagent. Total RNA was subjected to purification to isolate high-quality mRNA using poly-T attached magnetic beads (Invitrogen, USA), followed by fragmentation. The fragmented mRNA was then employed for the synthesis of first-strand cDNA utilizing random hexamer primers. This process was subsequently followed by the synthesis of second-strand cDNA. The library was prepared after end repair, A-tailing, adapter ligation, size selection, amplification, and purification. Paired-end 150-bp RNA-seq was performed on an Illumina platform. The quality of the raw reads was evaluated using Fastp (v.0.14.1) [50]. The expression levels quantification of each transcript was performed using Salmon v.1.9.0 [51] against the TAIR10 *Arabidopsis* reference genome. The transcripts per million (TPM) were then obtained. The downstream differential expression analysis was conducted in the R v.4.1.3 environment (http://www.r-project.org/). Differentially expressed genes (DEGs) were calculated using the DESeq2 package in R [52]. Selected DEGs were validated by RT-qPCR using primers from Supplementary Table S10. Gene Ontology enrichment analysis was performed using the clusterProfiler package in R [53].

### Statistical analysis and visualization

Raw data of 16S rRNA gene amplicon sequencing were processed using QIIME2 v.2022.2 [37]. All other statistical analyses were conducted using R v.4.1.3 (http://www.r-project.org/). The *vegan* package in R was used to assess the *alpha* and *beta* diversity of all the microbiome samples [54]. The Shannon and richness indexes were computed using the *diversity* function. Distance matrices based on the Bray–Curtis dissimilarity method for all samples were also generated. Permutational multivariate analysis (PCoA) of variance was performed to assess the effects of ecotypes, and treatment on variations in microbiome composition [54]. Permutational multivariate analysis of variance (PERMANOVA) was performed using the *adonis* function with 999 permutations. Differences in the relative abundance of specific phyla and genera between drought treatments within each ecotype were assessed using two-sided t-tests. Differences in the relative abundance of specific genera between drought treatments and within drought-tolerant or -sensitive ecotypes were assessed using two-sided Wilcoxon rank-sum tests. Statistical analyses were considered significant at *P* < 0.05. We use the *microeco* package in R to identify the biomarker genera of drought-tolerant and -sensitive ecotypes based on the randomForest [55, 56].

The NetShift analyses were conducted and visualized on the website https://web.rniapps.net/netshift. Phylogenetic tree of differential ASVs were built using FastTree v.2.1 [57], and were further visualized by using the *ggtree* package in R [58]. The heatmaps were generated using the *ComplexHeatmap* package in R [59]. The *UpsetR* package in R was used to generate upset plots [60]. The locations of the collection sites of natural *Arabidopsis* ecotypes, including longitude and latitude, were visualized using ArcGIS v.10.5 (Esri, Redlands, California, USA). The annual precipitation and annual mean temperature-related parameters that were used for correlation analysis with drought resistance index were extracted based on the WorldClim dataset [61]. All the plots in the present study were generated with *ggplot2* package in R-Studio, except for any special specification.

## Results

### Natural variations of drought tolerance among worldwide natural *Arabidopsis* ecotypes

**Figure 1:**
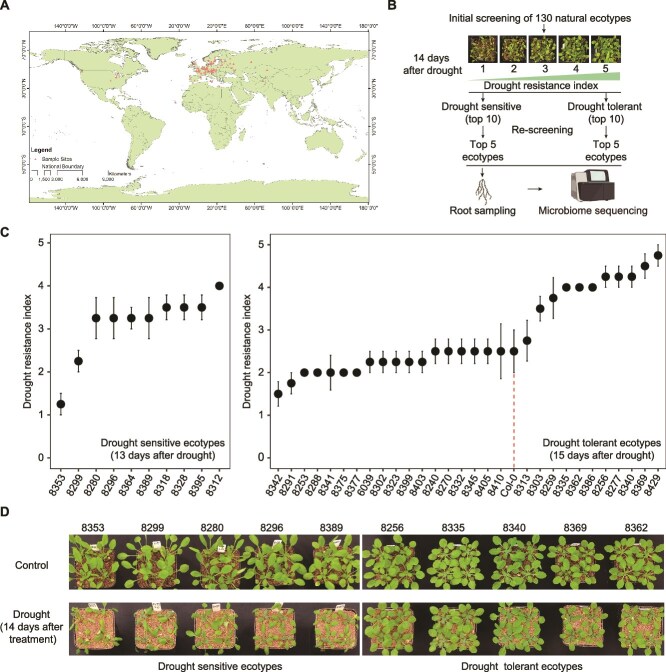
Variations of drought tolerance among natural ecotypes. (A) Diagram shows the original collection sites (red triangles) of all ecotypes used in the present study. (B) Experimental design for screening ecotypes with different levels of drought tolerance. (C) Characterization of drought-tolerant and -sensitive ecotypes based on the drought tolerance index. Due to the variations of drought tolerance, drought sensitive ecotypes were scored at 13 days after drought treatment, while drought tolerant ecotypes were scored at 15 days after drought treatment. The drought resistance was estimated based on the wilting phenotypes in B. (D) Phenotypes of the drought sensitive and tolerant ecotypes selected for microbiome analysis. Pictures were taken at 14 days with and without drought treatment.

To characterize the differences in plant drought tolerance of natural ecotypes, we performed phenotypic screening of drought tolerance among 130 representative *Arabidopsis* ecotypes from around the world (Fig. 1A). We collected natural soil from tropical rainforest (high microbial diversity) and dry-hot valley ecosystems (drought adapted microbiome) in Yunnan province, China (Methods). Both soil-borne pathogens and drought stress cause plant wilting phenotypes, and people usually determine disease index by estimating the wilting phenotypes [62]. We thus estimated a drought tolerance index using this phenotypic screening approach, based on the wilt degree (5, no wilting; 4, < 25% wilted leaves; 3, 25%–50%; 2, 50%–75%;1, 75%–90% mostly wilted), to reflect the damage of drought stress on natural *Arabidopsis* ecotypes (Fig. 1A, B).

During the initial screening, we conducted four batch experiments and assessed the drought tolerance index of a total 130 ecotypes to identify the extremely drought -tolerant and -sensitive ecotypes from each individual batch of experiments (Supplementary Table S1). From each batch experiment, we selected the top five tolerant and top five sensitive ecotypes for second round re-comparing. Thus, the top 20 drought-tolerant and top 20 drought-sensitive ecotypes were selected from the initial screenings and then grown together for a second-round screening (Fig. 1C; Supplementary Table S2). Based on the second-round screening, we identified the top 10 drought-tolerant (wilted at 15 days drought treatment) and top 10 drought-sensitive ecotypes (wilted at 13 days after drought treatment) (Fig. 1C). We performed a correlation analysis between temperature and precipitation-related parameters from ecotypes’ origin sites and their drought resistance (Supplementary Fig. S1). We found that ecotypes’ drought tolerance is negatively correlated with annual mean temperature. Considering that most selected ecotypes are from cold area with an annual mean temperature from 5–15 degree (Supplementary Fig. S1), this result indicates ecotypes that more adapted to cold stress also show stronger drought tolerance. This is probably because that both drought and cold stress cause dehydration and temperature changes, and there are convergence of stress-responsive genes which enhance tolerance to both stresses [63]. In contrast, drought tolerance indexes are not correlated with annual precipitation indexes, probably because most tested ecotypes are not from arid area (annual precipitation >500 mm) and do not cause drought stress for preadaptation. Collectively our data indicates the original sites might affect plant abiotic stress tolerance during adaptation and evolution.

Soil autoclaving can largely deplete natural soil microbiome and had been broadly used to confirm the existence of soil microbiome mediated regulation of host phenotypes [17, 64]. To dissect whether the high drought tolerance observed in drought-tolerant ecotypes is linked to a root microbiome-mediated drought protection effect, we compared the differences in drought tolerance between ecotypes growing in sterilized and non-sterilized natural soils. We calculated the relative fresh weight (the shoot fresh weight of drought-treated plants relative to the average shoot fresh weight of water control) as a proxy of plant drought tolerance of each ecotype, which indicates the loss of plant biomass due to drought damage. To further compare the differences in microbiome-mediated drought protection among these ecotypes, we assessed the microbiome-mediated drought protection effects by comparing the relative fresh weight ratios in sterilized and non-sterilized soils (Supplementary Fig. S2). Significantly, we observed varying levels of drought tolerance changes among different ecotypes in sterilized versus non-sterilized soils. For instance, ecotypes 8335 and 8369 exhibited much higher drought tolerance in non-sterilized soil compared to sterilized soil (Supplementary Fig. S2A), indicating a strong microbiome-mediated drought protection effect. In contrast, ecotypes 8303 and 8277 showed very weak difference in drought tolerance between non-sterilized and sterilized soils (Supplementary Fig. S2A). Consequently, we selected five ecotypes with the highest microbiome-mediated drought protection effects for further analysis (Supplementary Fig. S2B), alongside the top 5 most sensitive ecotypes for comparative microbiome studies (Fig. 1D).

### Distinct root microbiome composition in drought tolerant and sensitive natural *Arabidopsis* ecotypes

To characterize the root microbiome changes in the selected 10 ecotypes under drought stress, we performed 16S rRNA gene amplicon sequencing (V3-V4 region) for root samples of all ecotypes and bulk soil under both control and drought treatment. Firstly, the Bray–Curtis distance-based PCoA and PERMANOVA revealed that microbial community composition differed across different ecotypes (Supplementary Fig. S3). The plant ecotypes explained the largest proportion of the variation (*R*^2^ = 0.22, *P* < 0.001) followed by treatment (*R*^2^ = 0.09, *P* < 0.001), confirming that natural ecotypes contribute to variations in root-microbiome interactions. Additionally, we detected more differentially abundant (DA) phyla (among the top 10 phyla) in drought-tolerant ecotypes (9/10) compared to drought-sensitive ecotypes (5/10) (Supplementary Fig. S4A; Supplementary Table S3). Furthermore, we analyzed the genus-level composition to elucidate the influence of ecotypes on the root microbiome with higher taxonomic resolution. Similarly, compared to control group, 10 DA genera were detected in drought-tolerant ecotypes in the top 20 abundance-ranked list under drought condition, while only six were detected in drought sensitive ones (Supplementary Fig. S4B; Supplementary Table S4).

**Figure 2:**
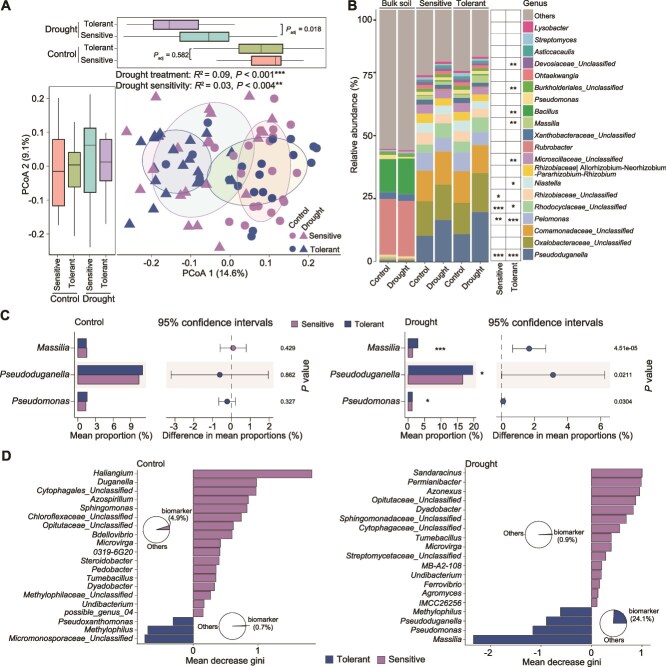
Drought-tolerant and -sensitive ecotypes differentially affect root microbiome changes upon drought stress. (A) Principal coordinates analysis (PCoA) was performed based on Bray–Curtis distance matrices of bacterial communities derived from the root microbiome samples of different types of ecotypes (PERMANOVA by Adonis). Box plots include the median (horizontal line inside the boxes), interquartile range (IQR) of 25th (lower edge of the box) and 75th (upper edge of the box) of Bray–Curtis distance-based values. *P* values were generated based on the Adonis pairwise comparison of microbial community composition between treatments and ecotypes with different drought sensitivity. (B) The relative abundances of major (top 20) bacteria genera in root microbiome from drought tolerant and sensitive groups. The asterisk (*) inside the right table indicates a significant difference (two-sided Wilcoxon rank-sum test) in the relative abundance of indicated genus between drought treatment and control groups in drought sensitive and tolerant ecotypes, respectively. (C) Three differential abundant genera between drought tolerant and sensitive ecotypes under drought stress were characterized. The graph shows the relative abundance of those genera in drought tolerant and sensitive ecotypes with and without drought stress. The asterisk (*) indicates a significant difference (two-sided Wilcoxon rank-sum test) between drought-tolerant and -sensitive groups. *, **, *** represent *P* < 0.05, *P* < 0.01, *P* < 0.001, respectively. (D) Prediction of biomarker genera for drought-tolerant and -sensitive ecotypes under control and drought conditions based on random forest analysis. Different colors indicate the ecotypes with different drought tolerance. The pie charts represent the cumulative relative abundance of all biomarker taxa in each group.

To assess overall community differences between drought tolerant and sensitive ecotypes, we merged the root microbiomes data from drought-tolerant ecotypes and sensitive ecotypes together as two groups. We observed significant differences between drought-sensitive and drought-tolerant ecotypes only under drought conditions (adonis pairwise, *R*^2^ = 0.05, adjusted *P* < 0.05), but not under control condition (*R*^2^ = 0.03, adjusted *P* = 0.58; Fig. 2A; Supplementary Table S5). Our PCoA analysis suggests that drought tolerant and sensitive ecotypes show slight but significant overall differences in microbiome beta diversity specifically under drought but not under control conditions. Furthermore, we analyzed the relative abundance of root microbial communities at the genus level (Fig. 2B). Nine differential abundant genera (among the top 20) were detected as significantly changed genera after drought stress in drought-tolerant ecotypes, whereas only four changed in drought-sensitive ecotypes (Fig. 2B). Among these, some genera showed a co-enrichment (like *Pseudoduganella*) or co-depletion (like *Pelomonas*) patterns in both drought tolerant and sensitive ecotype groups, while other taxa such as *Massilia* and *Bacillus* were only enriched in the drought-tolerant ecotypes (Fig. 2B). We further compared the differential abundant genera between drought-tolerant and -sensitive ecotypes. We only identified three highly abundant genera, including *Pseudoduganella*, *Massilia*, and *Pseudomonas*, were enriched in drought-tolerant ecotypes (Fig. 2C).

We further predicted biomarker taxa at genera level within each group based on RandomForest method. We discovered that drought-tolerant ecotypes exhibited fewer biomarker genera (3 biomarkers under control and four under drought conditions) compared to drought-sensitive ecotypes (17 under control and 15 under drought conditions; Fig. 2D). We also calculated the cumulative relative abundance of biomarker genera in each group. In drought-tolerant ecotypes under drought stress, biomarker taxa collectively accounted for 24.1% relative abundance (Fig. 2D), which is much higher than that in other groups (below 5%). Collectively, these results indicate that drought-tolerant ecotypes tend to enrich fewer biomarker taxa but with much higher cumulative abundance compared to the sensitive ecotypes. This further confirmed the differences in microbiome reshaping strategies between these two types of ecotypes.

### Existence of core keystone taxa driving microbiome network re-assembly upon drought

**Figure 3:**
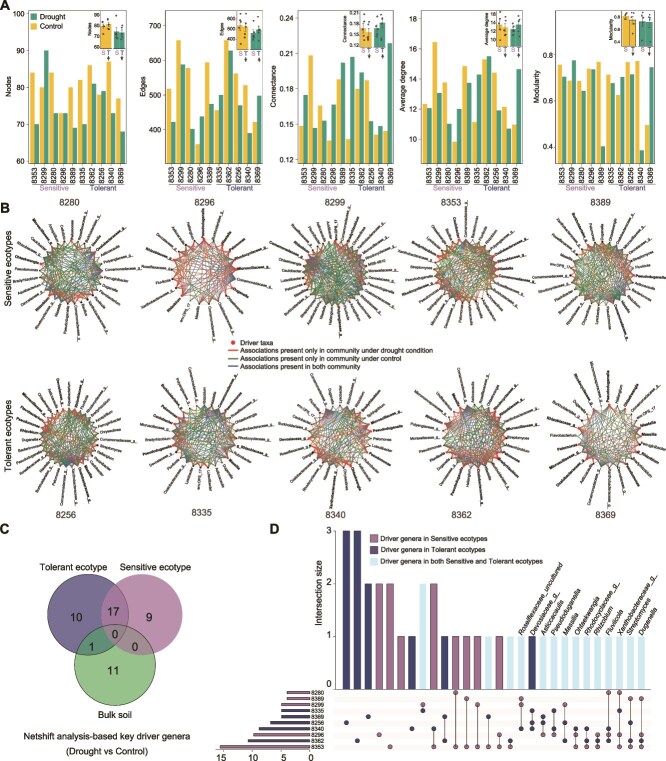
Microbial co-occurrence network changes in drought tolerant and sensitive ecotypes. (A) Comparisons of network nodes, edges, connectance, average degree, and modularity of the root microbiome in each ecotype under control and drought conditions. Bar plot (top right) within each panel diagram compares the corresponding network properties in each ecotype. Colors represent different treatments. (B) The potential “driver genera” for microbiome co-occurrence networks of each ecotype from control to drought treatment. The node name ending with "g" represents that the genus was not annotated within the specific taxonomic level, such as order or family. Node sizes are proportional to their scaled neighbor shift (NESH) score (a score for identifying important taxa within microbial associated networks). Nodes colored red represent the driver genera from control to drought treatment. Lines colored red, green or blue indicate node connections present only in the microbiome under drought, control or both conditions respectively. (C) Venn diagram showing the overlap of driver genera among drought tolerant, sensitive ecotypes and bulk soil groups. Number of driver genera of each group is indicated in the Venn diagram at the top of the panel. (D) Upset diagram showing the number of intersecting driver genera among ecotypes. Vertical bars with different colors represent the driver genera observed in different ecotypes.

Given that microbiome network analysis can offer more comprehensive insights into network interactions and identify keystone species driving microbiome reassembly processes [65, 66], we constructed co-occurrence networks based on Spearman’s correlations between paired ASVs in the root microbiome of each ecotype. We observed that drought stress generally reduced the size of the network (number of nodes and edges) in both drought-tolerant and sensitive ecotypes (Fig. 3A; Supplementary Table S6). Network connectance and average degree, which are widely used to characterize the network complexity, increased in most drought-tolerant ecotypes following drought stress, while decreasing in most drought-sensitive ecotypes (Fig. 3A). Furthermore, we also calculated the modularity and cohesion of networks, which can reflect network stability [67, 68]. We did not detect drastic changes or consistent trends in the modularity of different networks. However, most drought -tolerant ecotypes showed a decrease in total cohesion and negative cohesion (Supplementary Fig. S5). These results indicate that drought stress typically decreases network size across most ecotypes. However, drought-tolerant ecotypes exhibit higher network connectivity and complexity, and decreased cohesion upon drought compared to sensitive ecotypes.

To investigate the potential key driver taxa responsible for driving network changes from control to drought treatment, we conducted “NetShift” analysis on the co-occurrence networks between treatments. This approach allows the identification of keystone taxa by analyzing differences in interactions between pairwise networks [43]. We then identified a total of 37 driver genera across 10 ecotypes in response to drought (Fig. 3B, C; Supplementary Table S7), with 17 shared driver genera identified in both drought-tolerant and sensitive ecotypes. Only one driver genus was shared between the root microbiome and bulk soil microbiome under drought stress, supporting the influence of plant effects on shaping microbiome changes during drought (Fig. 3C). These results strongly suggest that drought stress triggers conserved core network changes among ecotypes, independent of their drought tolerance. Furthermore, we identified 12 taxa as drivers in more than three ecotypes (Fig. 3D), considered as potential core driver taxa under drought stress. Majority of these core driver taxa were found in both drought-tolerant and -sensitive ecotypes (Fig. 3D), confirming shared core network driver taxa between these groups. These taxa include the well-characterized drought-alleviating genus *Streptomyces* [25, 28], highlighting its critical role as a driver genus in network changes during drought stress. In summary, our results revealed a relatively overlapping and potentially conserved set of driver taxa driving network changes among drought tolerant and sensitive ecotypes.

### Existence of core drought responsive ASVs in response to drought

**Figure 4:**
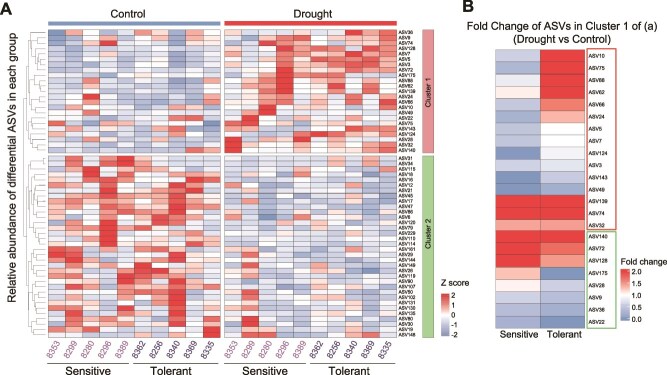
Core drought responsive ASVs among different ecotypes under drought stress. (A) The heatmap indicates the abundance of DA ASVs that were significantly enriched or depleted under drought treatment relative to the control. The heatmap is colored by normalized Z scores derived from the mean values of the relative abundance of each ASV in each sample. Cluster1 (colored by red) and Cluster2 (colored by green) show ASVs that are enriched or depleted in response to drought, respectively. Colored squares behind each ASV number indicate ASVs that were significantly enriched or depleted in at least five ecotypes, these were considered core drought-responsive ASVs. (B) The heatmap indicates the fold changes in the relative abundance of core ASVs in cluster1. The red and green rectangles indicate the ASVs with higher or lower fold changes in drought-tolerant ecotypes compared to drought-sensitive ecotypes.

Because we detected many conserved driver taxa in both drought-tolerant and sensitive ecotypes in response to drought, we hypothesized that drought might trigger relatively conserved changes in the taxonomic compositions in both drought tolerant and sensitive ecotypes. To test this, we calculated the DA ASVs between drought and control conditions across all ecotypes. For each genotype, we compared DA (Wilcoxon rank sum test, *P* < 0.05) ASVs with an average relative abundance higher than 0.1%. We identified 23 drought-enriched and 34 drought-depleted ASVs in clusters 1 and cluster 2, respectively (Fig. 4A). Among the 23 drought enriched ASVs, 21 of them were enriched higher than 1.2 folds in at least five ecotypes, which supports the existence of conserved core drought responsive ASVs among different ecotypes (Fig. 4A).

We found that drought-sensitive and -tolerant ecotypes share substantial overlap in those core drought enriched and depleted ASVs (Fig. 4A). To further reveal the potential differences between drought-sensitive and -tolerant ecotypes in their interactions with core drought responsive microbes, we hypothesized that the relative enrichment fold changes upon drought might differ between drought-sensitive and -tolerant ecotypes. To test this, we merged all drought-tolerant and sensitive ecotypes into two groups and compared the fold changes of all drought-enriched ASVs in cluster 1 after drought stress. As expected, although different ecotypes showed similar taxonomy of core drought enriched ASVs, the fold changes of several ASVs differed between the drought-tolerant and sensitive groups (Fig. 4B). This is reminiscent of a previous study showing that different *Arabidopsis* ecotypes show different levels of beneficial *Pseudomonas* colonization in roots, and beneficial *Pseudomonas* can only confer growth-promoting and disease-antagonizing effects in the compatible ecotypes (which support a high level of *Pseudomonas* colonization) [36]. Our data indicate that drought tolerant ecotypes might be able to form more “compatible” interactions and have higher fold changes of core drought responsive ASVs upon drought.

### Core drought responsive ASVs with higher fold changes in drought tolerant ecotypes confer stress-alleviating effects

**Figure 5:**
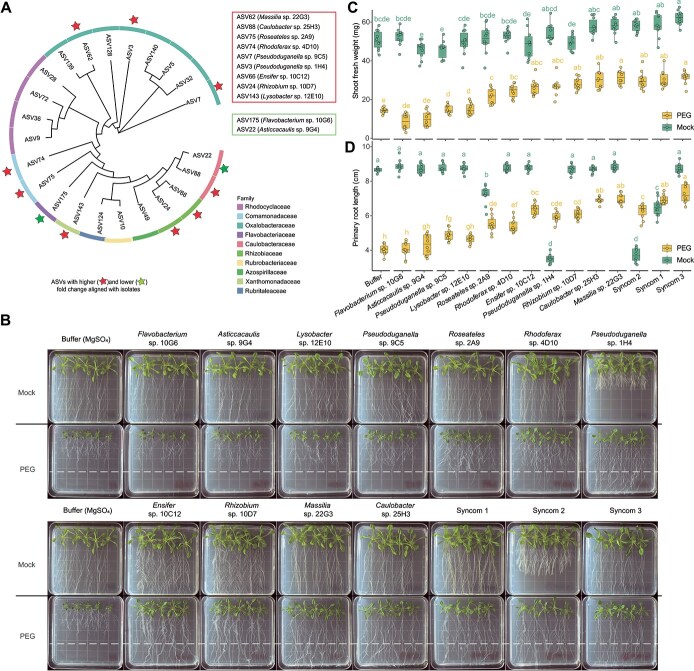
Validating the stress alleviating activities of core drought responsive strains. (A) Phylogenetic tree representing all core drought-enriched ASVs, color bars in the outer ring represent ASVs from different families. The red stars indicate AVSs that were successfully matched to our isolated strain collection. The red and green rectangles indicate that the average fold changes of relative abundance of these isolates are higher in drought-tolerant ecotypes and drought-sensitive ecotypes, respectively. (B) Growth phenotypes of plants grown on mock and PEG plates with different strains inoculations. Pictures were taken 21 days after germination. (C, D) Primary root length (C) and shoot fresh weight (D) of plants in B were assessed.

Among the 23 drought enriched ASVs in cluster 1 (Fig. 4A), 15 and eight of them show higher or lower average fold changes in drought-tolerant ecotypes compared to drought-sensitive ecotypes (Fig. 4B), respectively. We tried to validate the stress-alleviating activities of these strains in a reductionist approach. By utilizing a dilution-based high-throughput microbe isolation method [44], we recovered 1479 isolates and characterized 265 strains across seven phyla and 47 families based on 16S rRNA gene sequences [29]. Among these, we successfully aligned 11 of the above mentioned 23 core drought responsive ASVs for further functional validation (Fig. 5A; Supplementary Table S8). To accurately study the effects of individual strains on plant stress tolerance in mono-association, we established a gnotobiotic plate system for strain inoculation and PEG (Polyethylene glycol) induced osmotic stress treatment. We applied a 25% PEG overlay above normal 1/2 MS plates for 24 h to create PEG osmotic stress plates. Seedlings grown on PEG plates exhibited significantly compromised primary root growth and shoot growth defects, confirming the effectiveness of PEG-induced osmotic stress (Fig. 5B). Among the nine core drought-responsive strains that showed significantly higher fold changes in drought-tolerant ecotypes (Fig. 5A), all of them strongly promoted plant root and shoot growth under osmotic stress (Fig. 5B–D). In contrast, two ASVs [*Flavobacterium* sp. 10G6 (ASV175), *Asticcacaulis* sp. 9G4 (ASV22)], which showed lower fold changes in drought-tolerant ecotypes, either failed to protect or only very weakly protected plants from osmotic stress (Fig. 5B–D). To confirm the broad effect of core drought responsive strains on multiple host ecotypes, we chose two stress-alleviating microbes *Caulobacter* sp. 25H3 and *Massilia* sp. 22G3 for further tests. We found that both strains enhance plant growth in both drought-tolerant and -sensitive ecotypes (Supplementary Fig. S6). Overall, our data suggest that although core drought-responsive microbes are enriched in most tested ecotypes, for those strains with a higher fold change in drought-tolerant ecotypes tend to have stronger stress-alleviating activity.

To investigate potential synergistic interactions among the drought-alleviating strains described above, we tried to test the effect of synthetic microbial communities (SynComs) on plant stress tolerance. According to the drought protection effects of individual strain on plants, we chose the top 6 core drought responsive ASVs (ASV3, ASV7, ASV22, ASV24, ASV62, ASV66, and ASV88) for SynCom construction. A subset network analysis using the ASVs corresponding to these six bacterial strains across all samples was conducted to explore the interactions among them. Based on the two detected modules Supplementary Fig. S7), three SynCom inoculation strategies were designed: SynCom1, comprising all members of the six selected strains; SynCom2, a mixture of ASV3, ASV24, and ASV66 from module 1; and SynCom3, a mixture of ASV62, ASV74, and ASV88 from module 2 (Supplementary Fig. S7). We found that all three SynComs significantly promote shoot and root growth under osmotic stress (Fig. 5B–D). However, only SynCom1 exhibits strong drought protection effects in soil grown plants (Supplementary Fig. S8). That indicates strain interactions between these two modules might be critical for the stability and function of SynComs in the complex soil microbiome environment.

### Drought alleviating *Masslia* strain broadly reshapes stress responsive pathways in roots

**Figure 6:**
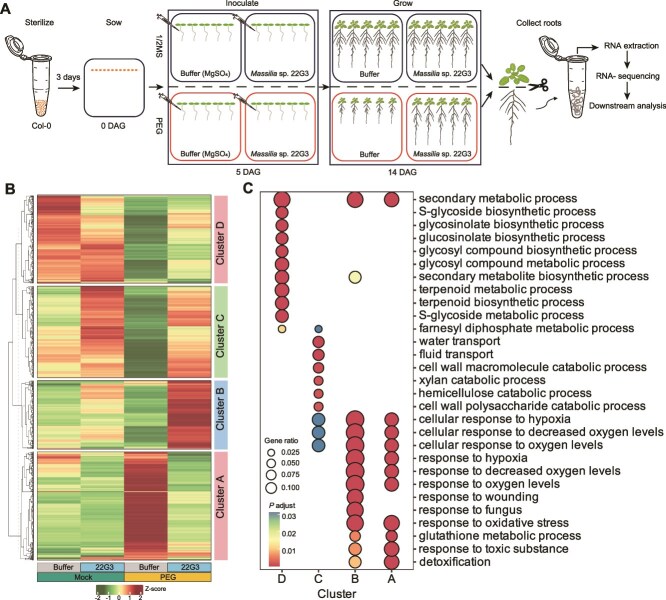
*Massilia sp.* 22G3 broadly modulates the growth- and stress-response-related pathways upon osmotic stress. (A) The schematic diagram shows the inoculation and sampling processes of transcriptome analysis. DAG: Days after germination. (B) K-means clustering of all differentially expressed genes among different treatments. The x-axis reports the groups treated with or without *Massilia* sp. 22G3 under mock or PEG plates. The heatmap is colored by normalized Z scores. (C) Illustration of top 10 enriched GO terms from each cluster in B. Each dot represents a GO term. The size of each dot indicates the number of genes associated with that GO term (GeneRatio) in each cluster in B. The color scale of the dot represents the adjusted *P* value.

Among the five core drought-enriched strains that show significant stress-protecting activity, we chose the *Massilia* sp. 22G3 strain for further mechanistic study because: (i) the *Massilia* genus is predicted to be the most representative biomarker species for drought-tolerant ecotypes under drought stress (Fig. 2D); (ii) the relative abundance of the *Massilia* genus is significantly higher in drought-tolerant ecotypes compared to the sensitive ones (Fig. 2C); and (iii) the *Massilia* genus is identified as a key driver genus for network changes in both drought-tolerant and sensitive ecotypes (Fig. 3D). We conducted RNA-seq study in root samples to survey the transcriptome landscape underlying the *Massilia* sp. 22G3-mediated stress-alleviating effects (Fig. 6A). We identified a total of 2036 DEGs (Supplementary Table S9) after PEG treatments with and without *Massilia* sp. 22G3 inoculation. We performed K-means clustering and GO enrichment analysis to distinguish different gene expression patterns affected by 22G3 inoculation and their potential functions (Fig. 6B).

**Figure 7:**
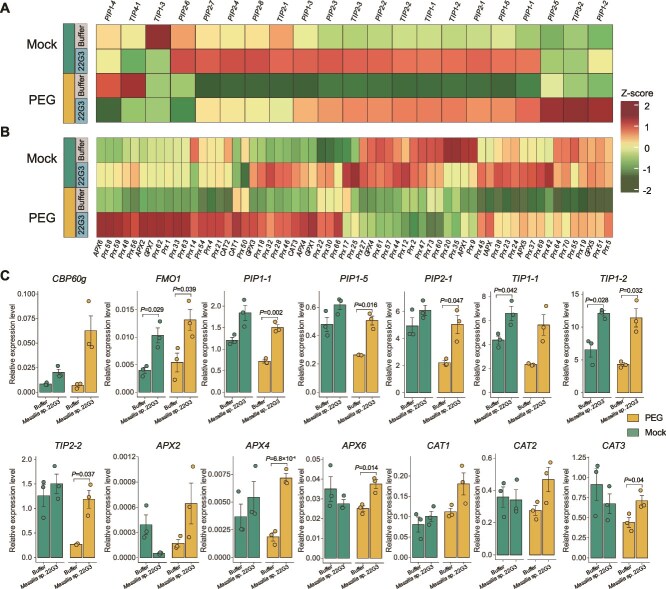
*Massilia* sp. 22G3 broadly promotes the expression of aquaporin and ROS scavenging genes upon osmotic stress. (A, B) Heatmaps illustrate the expression of aquaporin genes (A) and ROS scavenging gene expression (B) in roots inoculated with or without *Massilia* sp. 22G3. The heatmap is colored by normalized Z scores. (C) Validation of the expression of 14 marker genes related to immune response, aquaporin and ROS scavenging genes via qRT-PCR. Only the significant differences (marked with exact *P* values) between *Massilia* sp. 22G3 treatment and mock are shown (two-sided Student’s t test). Data represent mean (bar) ± standard error of the mean (error bar).

We found that almost all osmotic stress induced genes (cluster A) are substantially blocked by the inoculation of beneficial *Massilia* sp. 22G3. Genes in cluster A are enriched in the GO terms related to oxidative stress (Fig. 6C), detoxification and extracellular stimulus, including diverse stress related genes like *SENESCENCE-ASSOCIATED GENE 21* (AT4G02380), *Autophagy-related protein 8b* (AT4G04620), and *Heat shock protein 90–1* (AT5G52640) (Supplementary Table S9). This result indicates that *Massilia* sp. 22G3 inoculation broadly suppresses osmotic stress triggered transcriptome perturbations in roots, consistent with its strong stress protection phenotype in roots. We found that inoculation of 22G3 activates the expression of 524 genes in cluster C independent of stress treatment. Those genes are drastically enriched in the GO functions related to water/fluid transport and cell wall biosynthesis (Fig. 6C). This is further supported by the evidence that among 23 PIPs and NIPs, 20 of them show much higher expression after *Massilia* sp. 22G3 inoculation (Fig. 7A). This provides critical clue that *Massilia* sp. 22G3 may enhance root water up-taking activity to combat osmotic stress and maintain root growth under stresses. Moreover, inoculation of *Massilia* sp. 22G3 specifically up-regulate genes in cluster B only under osmotic stress but not mock condition, which might include microbes induced stress specific regulators to combat osmotic stress. As expected, we found multiple stress rescuing regulators or enzymes are involved in cluster B. For example, cluster B genes include *ATJUB1* (*NAC042*, At2G43000), which is a master regulator of oxidative stress tolerance and overexpression of this gene can strongly delay senescence and enhance tolerance to various abiotic stresses including drought and salt [69]. Consistent with this result, we found that diverse ROS scavenging enzyme genes are up-regulated (Fig. 7B). Moreover, defense related GO terms are also enriched in cluster B genes, and we found that some well-known root immune marker genes (AT1G18570-*MYB51*, AT2G30750-*CYP71A12* [70]; AT2G35980-*ATNHL10*; AT5G26920-*CBP60G*; AT2G19190-*FRK1* genes). It had been reported that plant immune responsive genes share some overlap with general abiotic stress responsive pathways [71]. Thus timely up-regulation of immune related genes by *Massilia* sp. 22G3 predominantly under stressed conditions may collectively reshape the stress responses in roots. Our qRT-PCR data further validated the expression patterns of key stress or immune responsive genes detected by RNA-seq (Fig. 7C). Collectively, our study provided comprehensive mechanistic insights underlying beneficial *Massilia* sp. 22G3 mediated stress-alleviating process, which are mainly related to the enhanced expression of water up-taking proteins and ROS scavenging enzymes, as well as timely activation of immune and stress responsive genes under stress condition in roots.

## Discussion

Plants are sessile organisms that are frequently exposed to diverse biotic and abiotic stresses, and diverse stresses also shift plant associated microbiomes and cause enrichment of potential stress-alleviating microbes [18, 72, 73]. Microbiome engineering has the potential to promote second green evolution in agroecosystems [74]. However, a big challenge is that beneficial strains usually fail to protect crops in diverse local environments due to differences in plant genotypes and soil types. Pioneering studies have utilized natural ecotypes to characterize the "core microbiome" in *Arabidopsis* [4]. Although currently there are increasing interests in characterizing and application of “core microbiomes” in crops [3, 5], very few studies focused on the “core microbiome” changes under agricultural relevant stresses. Since certain stress-alleviating microbiomes tend to be enriched or potentially “recruited” under both biotic and abiotic stresses, theoretically, revealing the key microbial ecological changes upon stresses can also guide the discovery of stress-alleviating microbes. In this work, we can compare the “core stress responsive microbiome” changes among natural ecotypes and confirmed that can provide predictions of conserved keystone taxa changes relevant for plant growth and stress tolerance, as well as agriculture applications in the future.

By integrating comparative microbiome studies, high-throughput culturing, and functional assays, we systematically characterized the conserved core microbiome changes among natural ecotypes and their relevance to plant stress tolerance. We found that drought -tolerant and -sensitive ecotypes show differences in the beta diversity, DA taxa, and network interactions, indicating that the variations of root-microbiome interactions might contribute to stress adaptations in different ecotypes. We found that both drought tolerant and sensitive ecotypes share substantial overlap in the ASV compositions of “core drought responsive microbiome”. Although drought-tolerant and drought-sensitive ecotypes share similarities in the composition of the core drought-responsive ASVs, these strains in drought tolerant ecotypes with higher fold changes tend to have stronger stress-alleviating activity (Fig. 5B, C). Our data indicates that, although drought stress triggers conserved core microbiome changes in both drought-tolerant and sensitive ecotypes, the relative fold changes of these strains in drought tolerant ecotypes can be used to predict their stress-alleviating activities. Furthermore, microbial interaction network analysis identified hub species, which are highly interconnected and play critical roles in shaping community structure and function [75]. Our work also revealed the shared keystone taxa driving network shifts among ecotypes.

Although Actinobacteria or *Streptomyces* is one of the best-characterized drought-alleviating taxa show conserved enrichment in different plant species across broad phylogenetic distances [21, 24, 28], this trend seems to be soil type dependent and was not observed in our study and a lot of other studies [25, 76–78]. Even though our work identified *Streptomyces* as a driver genus influencing microbiome network shifts in four of the 10 tested ecotypes upon drought stress (Fig. 3D), indicating that *Streptomyces*’ network importance can be enhanced even it is relative abundance is not enriched in some soils. Although the enrichment of Actinobacteria represents one of the conserved drought responsive microbiome changes in different species, using different plant species might cause huge basal level microbiome differences and limits the accurate discovery of more “species specific drought responsive microbiomes”. As expected, we used ecotypes from the same plant species and successfully identified more “core drought responsive” microbes from several different families, which could be important agricultural relevant beneficial strains for specific species or soil types.

Over the past decade, the rapid development of culture-independent microbiome composition and function studies has broadly described the associations between microbiota and plant health [1]. However, there remains an urgent need for deeper mechanistic studies underlying root-microbiome interactions to advance functional microbiome research. Actually it had been estimated that 10 model microbe species comprise half of the bacteriology literature [79]. Plant root associated microbiome is one of the most diverse microbial communities on this planet, and a lot of model plants or crops are genetically and genomically trackable. Thus plant-microbiome interaction systems can be used to delve into the molecular and genetic mechanisms governing microbes mediated growth promoting and stress tolerance. It had been proposed that root associated microbes might promote drought tolerance by producing exopolysaccharides [80–83], or manipulating root hormone signaling [84, 85] and water loss [86]. Our recent study provided genetic evidence in bacterial that proline metabolizing genes might affect oxidative stress tolerance and drought alleviating effects in Rhizobiaceae [29]. In this work, our transcriptome analysis further revealed the broad effect of *Massilia* sp. 22G3 on the expression of immune, ROS scavenging and water up-taking genes under either mock or PEG stress. In the future, it is worth further studying the detailed genetic or biochemical mechanisms of drought protection in *Massilia* sp. 22G3, which would help develop beneficial microbes-based strategies to enhance plant stress tolerance.

In the present study, *Massilia*, a genus that belongs to the family of Oxalobacteriaceae, was identified as a biomarker taxon which was significantly enriched after drought stress. This genus has emerged as a pivotal plant-beneficial microbial genus. For example, it has also been reported to promote plant nitrogen uptake [87]. Recent study also suggests that *Massilia* strains can act as a keystone taxon critical for root development especially lateral root formation under low-nitrogen stress [88]. In our study, we characterized the *Massilia* sp. 22G3 strain from the *Massilia* genus, which almost totally recovered primary root growth on PEG plates. Moreover, *Massilia* species are frequently isolated from extreme environments such as desert [89, 90] and rocks [91]. The broad environmental distribution and plant stress-alleviating activity of *Massilia* make it a promising target for engineering plant microbiomes under diverse abiotic stresses. In the future, developing genetic and genomic tools in the *Massilia* will help deepen our understanding of the molecular dialog mechanisms between roots and *Massilia* members. Additionally, it would be promising to develop *Massilia*-based probiotic strains or synthetic communities to promote crop growth and stress tolerance.

## Supplementary Material

Supplementary_information_wraf067(1)

Supplementary_table_S1-S10_wraf067

## Data Availability

The raw 16S rRNA gene amplicon sequencing data in this study have been deposited in the [Genome Sequence Archive in the BIG Data Center, Chinese Academy of Sciences] repository, [accession PRJCA023287: https://ngdc.cncb.ac.cn/gsa/browse/CRA014847], and the raw data for RNA-seq study is deposited under accession number CRA017120 [https://ngdc.cncb.ac.cn/gsa/browse/CRA017120].
