# Supplementary material for: Identification of stress-alleviating strains from the core drought-responsive microbiome of Arabidopsis ecotypes
Source: ISME J. 2025 Apr 9;19(1):wraf067. doi: 10.1093/ismejo/wraf067 (PMC12043206; doi:10.1093/ismejo/wraf067)
Supplement: Supplementary_information_wraf067(1) [file supplementary_information_wraf067(1).docx]

**
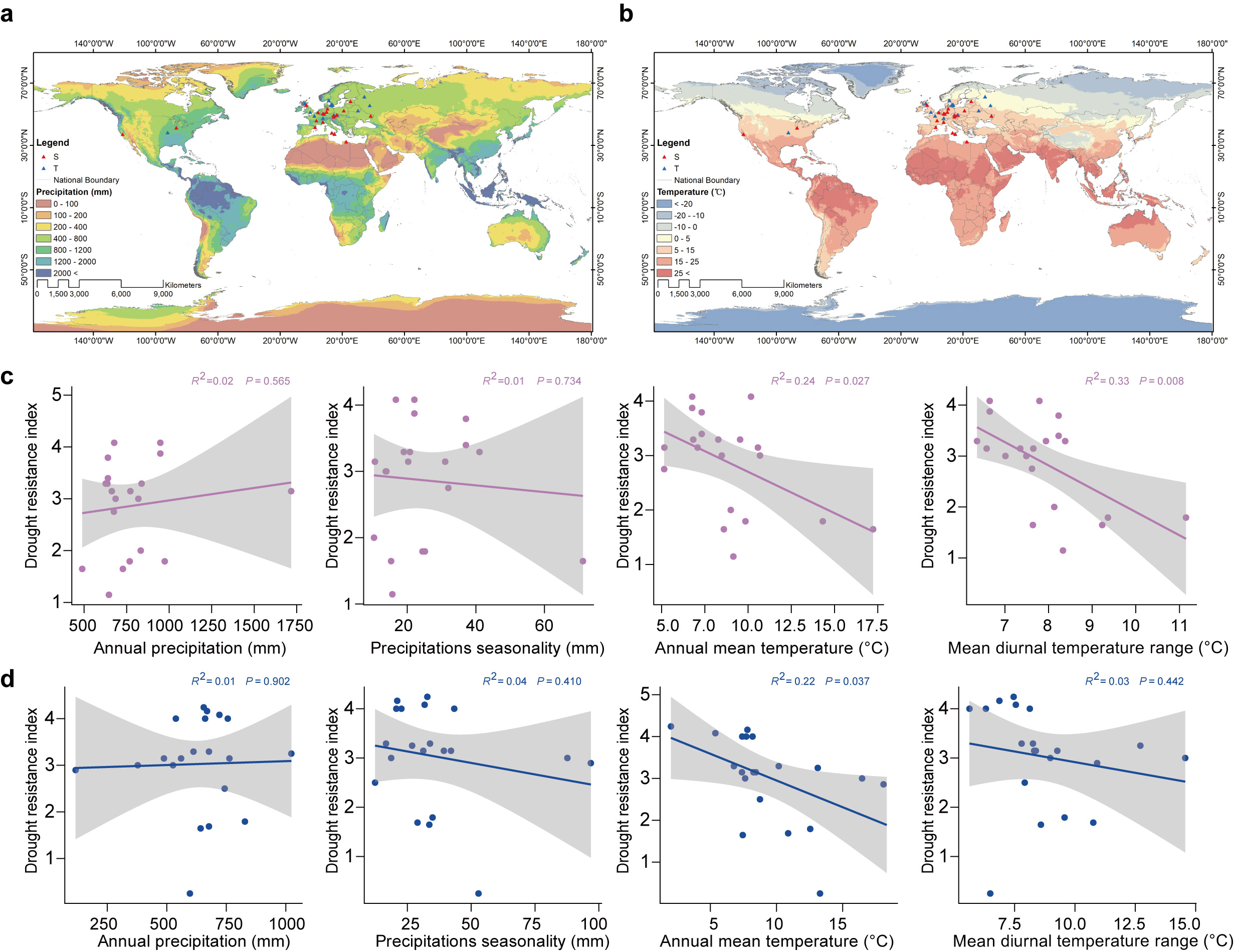
Supplementary Fig.1** Relationships between environmental origins and drought resistance of *Arabidopsis* ecotypes. **a-b** Global maps show the spatial distribution of ecotypes origins, as well as the annual precipitation (**a**) and annual mean temperature **(b)** of each location (*n*=20). **c-d** Correlation analysis (based on a linear regression model) of the precipitation and temperature-related parameters from each ecotype origin site and the corresponding drought resistance of specific ecotype (*n*=20 for each group). The *P* values and *R*^2^ values calculated from linear regressions are shown.


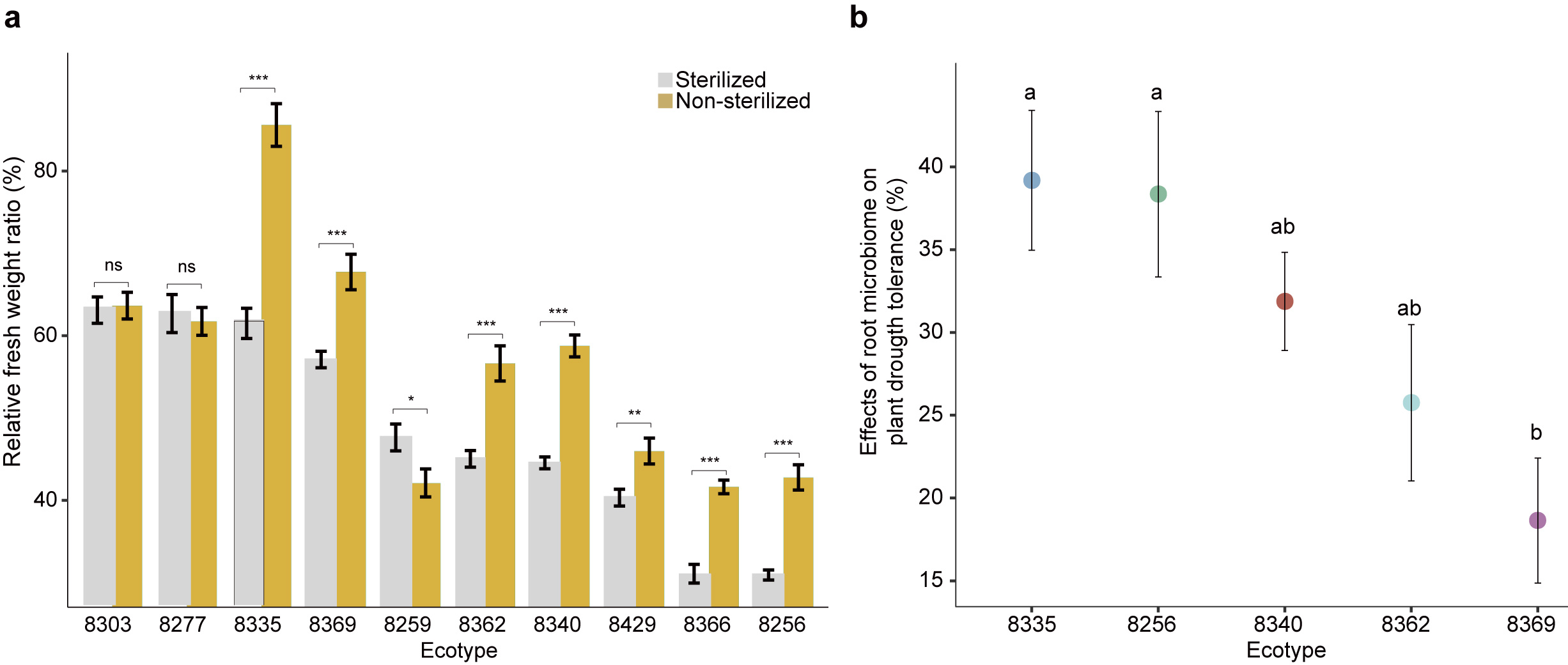


**Supplementary Fig. 2** Microbiome-mediated drought protection differs among *Arabidopsis* ecotypes. **a** The relative fresh weight (the shoot fresh weight of the drought-treated group relative to the average shoot fresh weight of the control group) of the top 10 most drought-tolerant ecotypes was measured at 14 days after withholding watering. Samples from both sterilized and non-sterilized soils were individually calculated. Values are presented as mean ± standard error of the mean. n=20 for each group. *, **, ***: *P* <0.05, *P* <0.01, *P* <0.001 (two-sided t-test), respectively. The x-axis labels represent the accession IDs of each ecotype. Different lowercase and uppercase letters indicate significant differences in relative fresh weight among ecotypes in sterilized and non-sterilized substrates, respectively. **b** Top 5 ecotypes with the strongest microbiome-mediated drought protection effects were selected. One-way ANOVA followed by LSD test.


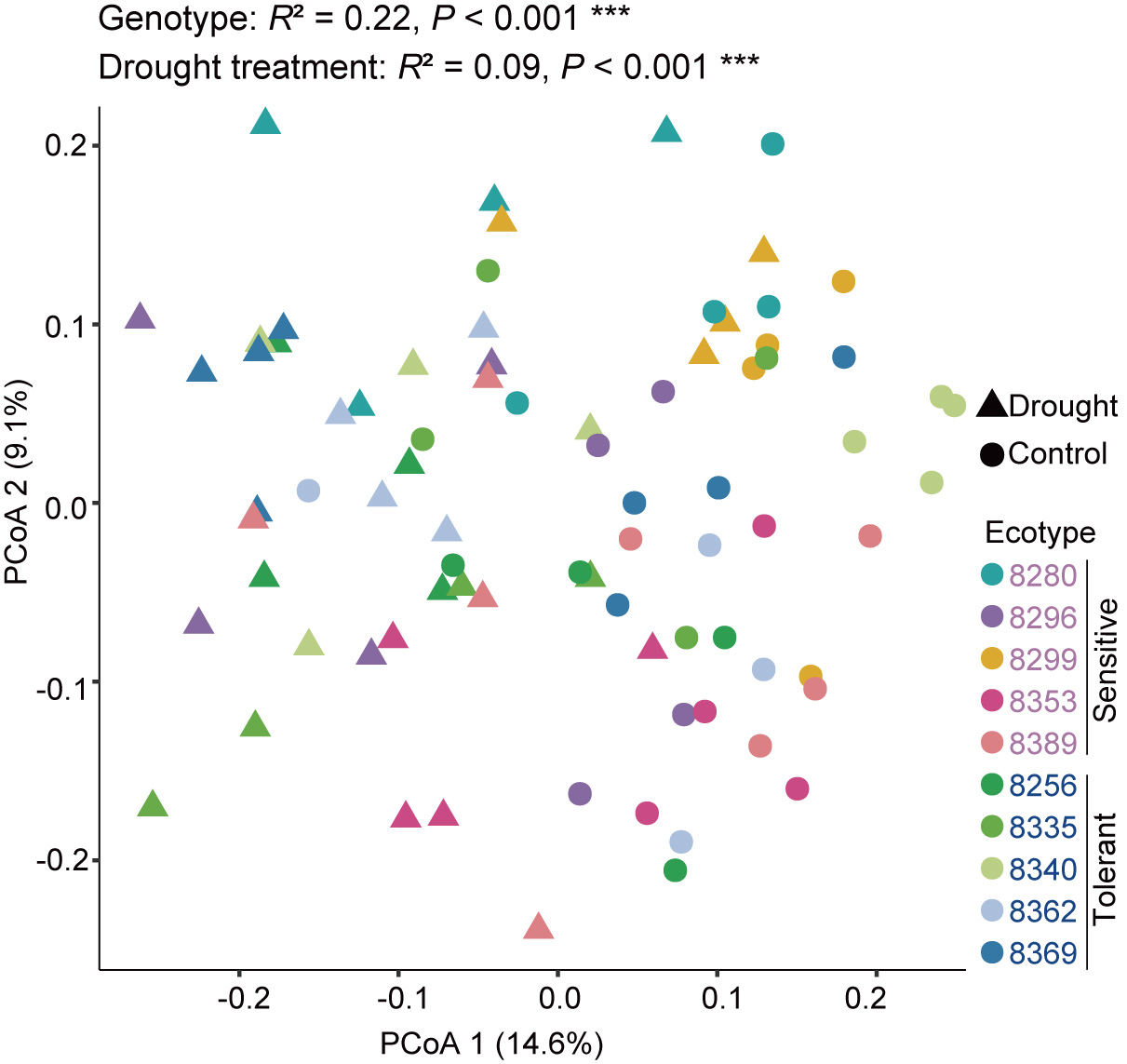


**Supplementary Fig. 3** Principal coordinates analysis (PCoA) was performed based on Bray–Curtis distance matrices of bacterial communities from the root samples of all ecotypes (PERMANOVA by adonis). Different colors indicate different ecotypes and shapes represent treatments.


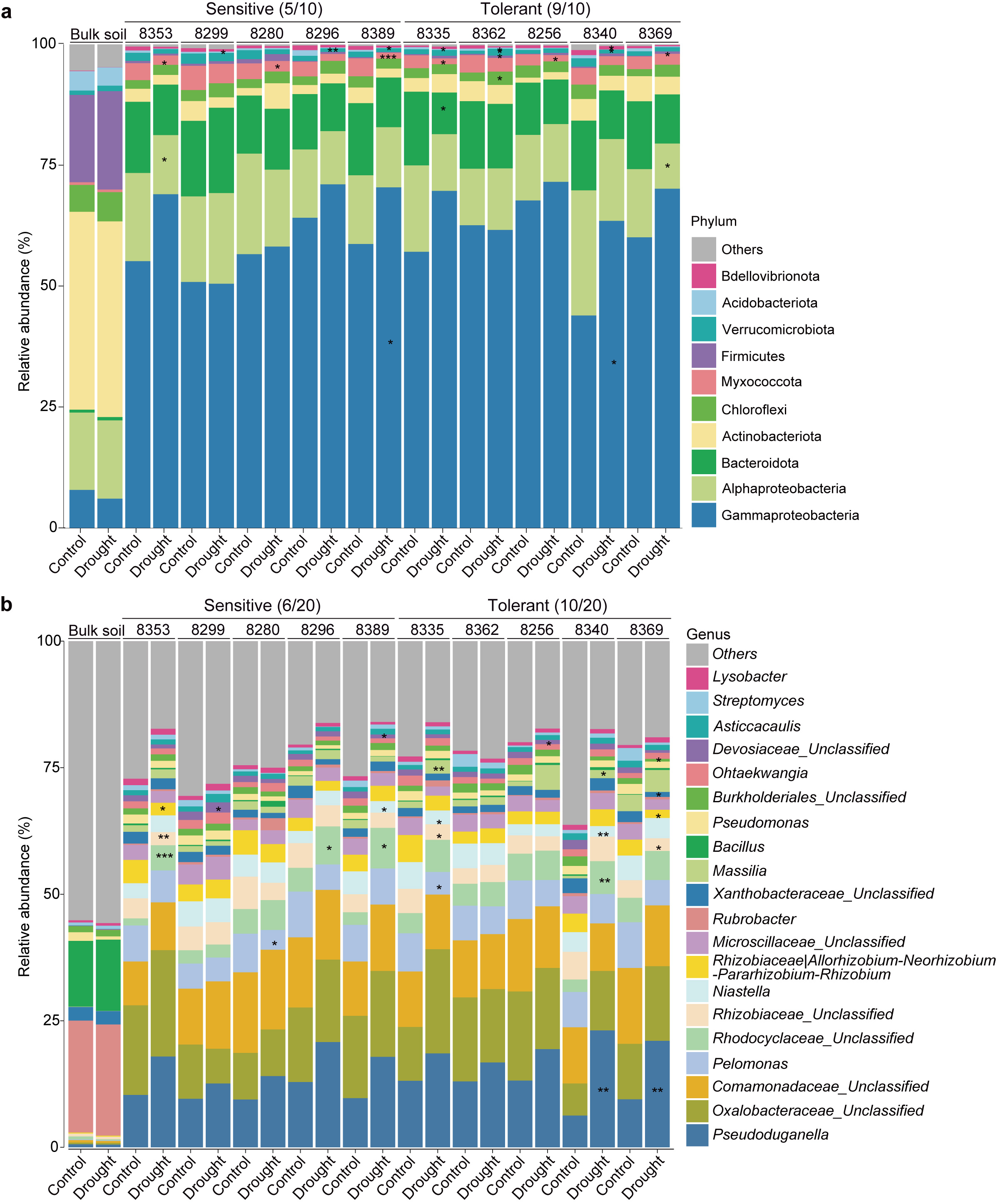
**Supplementary Fig**. **4** The relative abundance changes of phyla and genera in different ecotypes with and without drought treatments. **a-b** Relative abundance of the top 10 abundant phyla (a) and top 20 abundant genera (b) in all tested genotypes and conditions. n = 4 biological replicates. The asterisk (*) inside the bar plot indicates a significant difference (two-sided t-test) between drought and control groups within each ecotype. Drought tolerant and sensitive ecotypes were indicated at the top of the graph. The total numbers of differentially abundant taxa in drought tolerant and sensitive ecotypes were listed on the top. *, **, *** represent *P* <0.05, *P* <0.01, *P* <0.001, respectively.


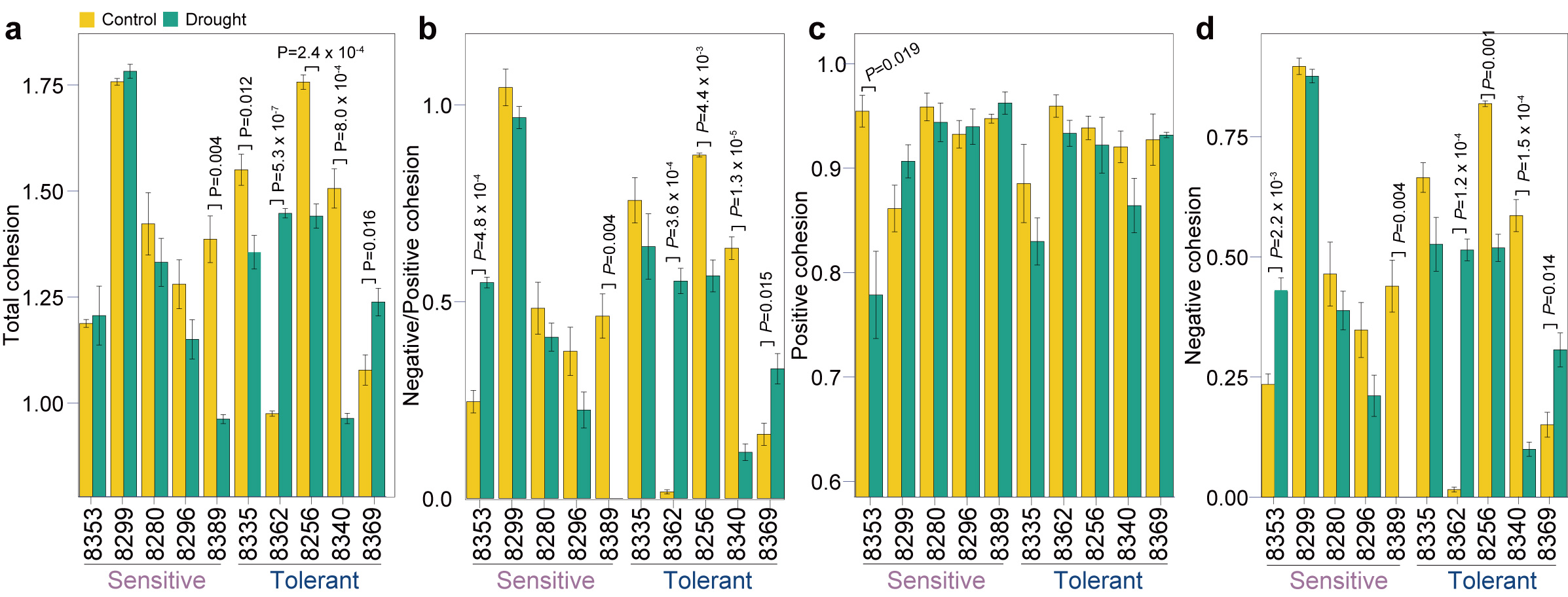
**Supplementary Fig. 5.** Cohesion changes in root microbial network of each ecotype across control and drought treatments. **a-d** Differences in (a) total cohesion (sum of positive cohesion and the absolute value of negative cohesion), (b) proportion of negative cohesion relative to positive cohesion, (c) positive cohesion, (d) negative cohesion of root microbial network of each ecotype across treatment group. Only the significant differences (marked with exact *P* values) between control and drought treatment are listed above each bar (two-sided Student’s t test). Data represent mean (bar) ± standard error of the mean (error bar).


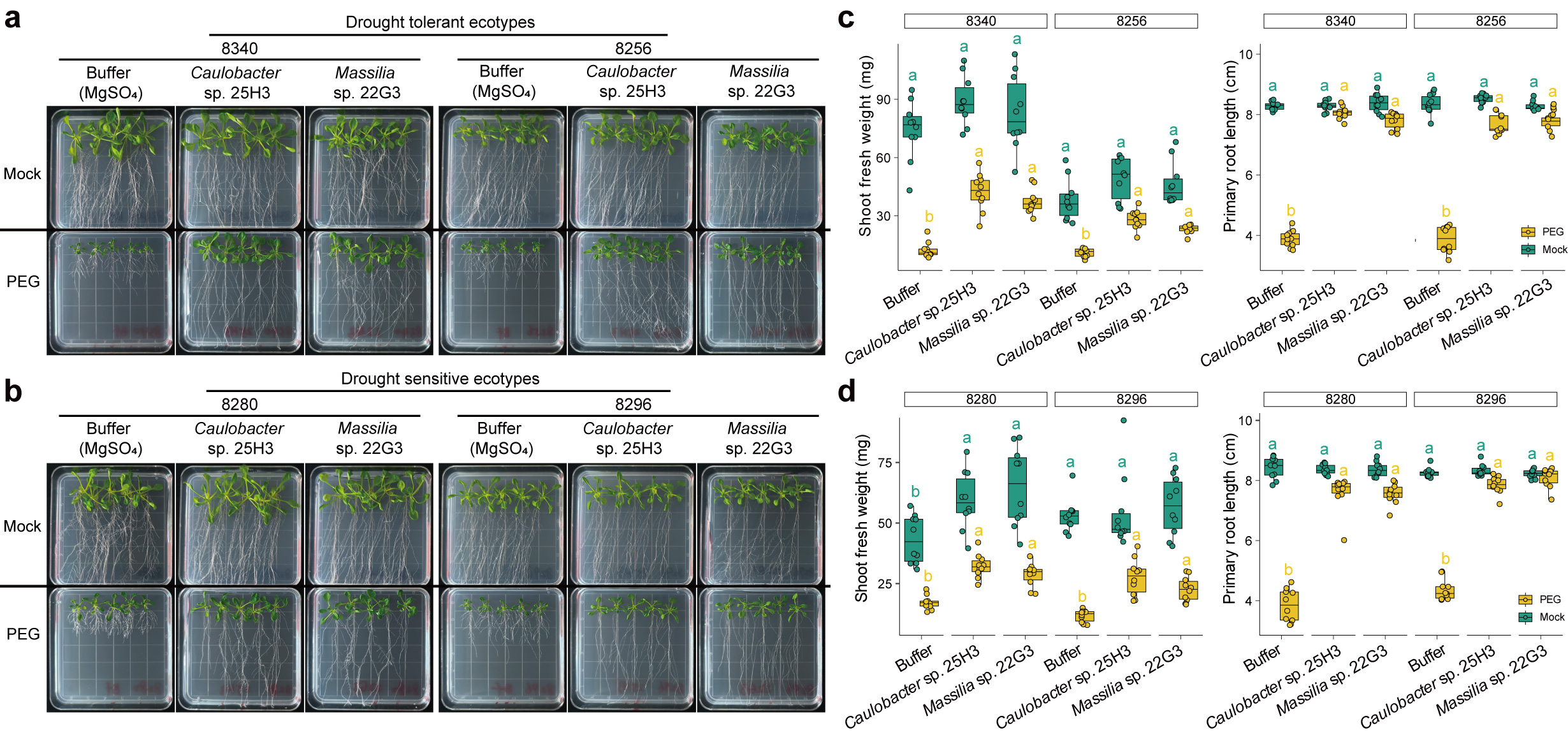


**Supplementary Fig. 6** Core drought responsive microbes confer broad stress-alleviating activities in both drought-tolerant and -sensitive ecotypes. **a-b** Pictures show the growth phenotype of drought tolerant ecotypes (8340, 8256) and sensitive ecotypes (8280, 8296) under mock and 25% PEG plates with and without microbial inoculation. Seedlings were transferred onto Mock and PEG plates at 5 days after germination, and bacteria were inoculated onto root surface at the same day. Pictures were taken at 21 days after germination. **c-d** Primary root length and shoot fresh weight were assessed for drought-tolerant ecotypes (c) and drought-sensitive ecotypes (d) grown on mock and PEG plates shown in a-b. Different lowercase letters, colored in green and yellow, indicate significant differences in growth phenotypes (shoot fresh weight or primary root length) of each ecotype within each treatment group (one-way ANOVA followed by LSD test, *P* values were corrected using Bonferroni method, n = 10).

**
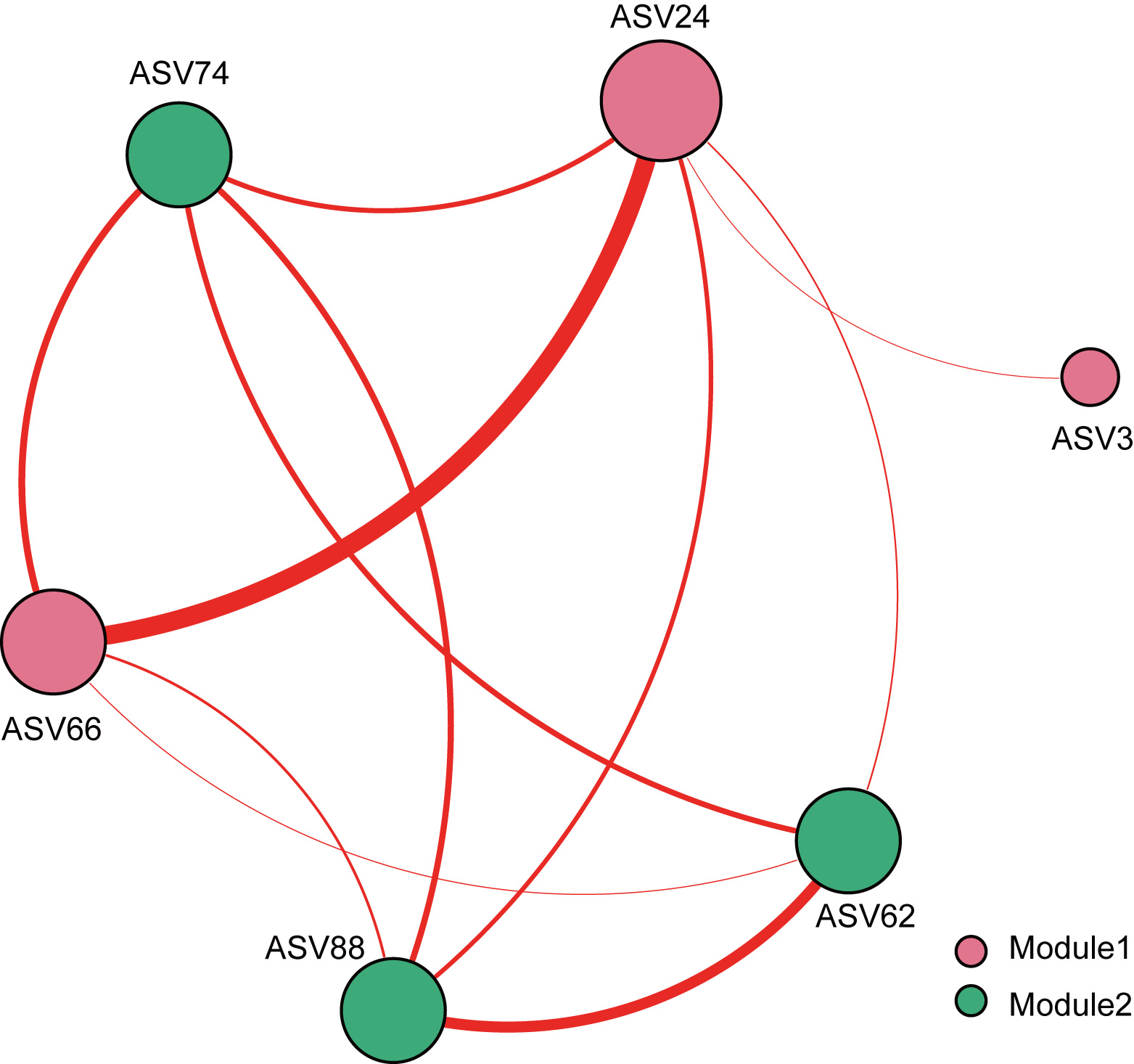
Supplementary Fig.7** Co-occurrence network of the top 6 core drought-responsive ASVs showing the highest drought protection effects. Each node indicates an ASV. The same color represents the nodes belonging to same module (calculated using igraph package in R). Edges represent significant correlations.


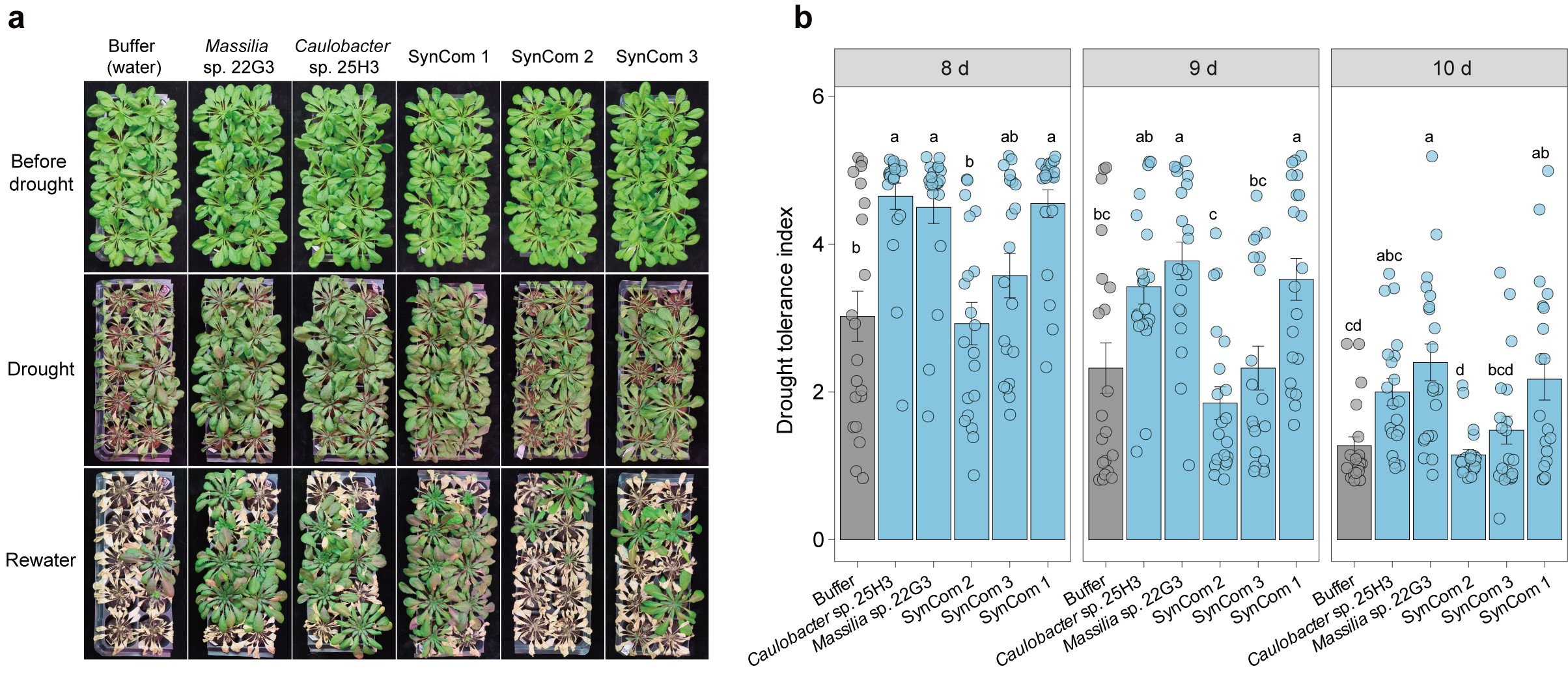


**Supplementary Fig. 8** Core drought responsive microbes and their SynComs exhibit significant drought alleviating activities in soil grown plants. **a** Pictures show plant phenotypes in soil after drought treatment (9^th^ day) and 7 days after re-watering. Plants were inoculated with the indicated microbes or Syncoms on the 21^st^ day after transplantation. **b** Time-series quantification of plant drought tolerance index changes. Values are presented as mean ± standard error of the mean. Different lowercase letters indicate significant differences in the drought tolerance index across different inoculation treatments within each day (one-way ANOVA followed by LSD test, *P* values were adjusted using Bonferroni method, n = 20).
